# Accidental Organophosphate Poisoning in a Patient Due to a Bed Bug Pesticide in the United Kingdom: A Case Report

**DOI:** 10.7759/cureus.75149

**Published:** 2024-12-05

**Authors:** David Griffiths, Jami Hanif, Jacob McCammon, Luke McMaster, Cameron Gemmell

**Affiliations:** 1 Anesthesiology, University Hospital Lewisham, London, GBR; 2 Anesthesiology, Queen's Hospital, London, GBR; 3 Emergency Medicine, Sunshine Coast University Hospital, Sunshine Coast, AUS; 4 Emergency Medicine, Cairns Base Hospital, Cairns, AUS; 5 Emergency Medicine, North West Regional Hospital, Burnie, AUS

**Keywords:** airway intubation, anti cholinergic storm, healthcare worker safety, multidisciplinary teamwork, muscle relaxant, organophosphate pesticide poisoning, personal protective equipment, total intravenous anaesthesia

## Abstract

Organophosphate (OP) compounds, developed during World War II, are a group of chemicals used as pesticides, insecticides and herbicides. As irreversible inhibitors of the enzyme acetylcholinesterase (AChE), they reduce anti-cholinesterase activity and therefore increase acetylcholine (ACh) levels at the neuromuscular junction (NMJ). Diazinon, the OP leading to the patient's symptoms in this report, is an amber-brown liquid that was once the most widely used insecticide in the United States of America. The patient inhaled diazinon and experienced a mixture of muscarinic and nicotinic receptor symptoms ranging from acute cholinergic syndrome to mild delayed intermediate syndrome. The patient was intubated, managed in the intensive care unit (ICU) with level 3 care, and made a full recovery. The report focuses on evolving toxicology advice and the adjustment of muscle relaxant dosing for intubation.

## Introduction

Organophosphate (OP) compounds are formed through esterification between phosphoric acid and alcohol. Their uses range from pesticides and herbicides to nerve agents in chemical warfare [1]. An example is diazinon, which was industrialised in 1952 by a Swiss chemical company [2] and became the most widely used pesticide in the United States of America. It was cancelled for residential use in 2000 by the US Environmental Protection Agency to reduce the risk of pesticides at home, especially to children [3]. Such acts have reduced the prevalence of OP poisoning in more developed countries, but it remains prevalent in less developed countries often presenting with non-accidental poisonings [4]. An increased interest in OP poisoning in the United Kingdom (UK) stems from recent poisonings such as the Skripel family in March 2018 in Salisbury [5]. In June 2022, the North Atlantic Treaty Organisation (NATO) identified that the deliberate use of chemical, biological, radiological and nuclear materials had the potential to “sow panic and strain national response capabilities” [6]. OP causes irreversible phosphorylation of the acetylcholinesterase enzymes leading to a spectrum of pathology, depending on the time and dose of the agent. These range from cholinergic crisis and intermediate syndrome to OP-induced delayed neuropathy and chronic OP-induced neuropsychiatric disorder [7]. The method of intoxication is by absorption through the skin, gut or bronchi following inhalation as in this case. The suggested management of OP poisoning involves atropine, pralidoxime (a cholinesterase reactivator), benzodiazepines and supportive care [8]. The evidence and advice regarding these treatments can vary and management of such patients is heterogeneous from country to country. Specifically, OP poisoning raises challenges in muscle relaxant dosing with limited literature to support a specific agent or dose.

## Case presentation

Patient background

A 40-year-old female weighing 70 kilograms presented to the Accident and Emergency (A&E) department. Her past medical history included ischaemic heart disease for which she took aspirin and atorvastatin, hepatitis B, vitamin D deficiency and lower back pain. She slept in a bedroom treated with diazinon for a bed bug infestation next to her husband; however, unlike her husband, covered her face with the bed sheet.

Day 0 of admission

05:00: The patient woke feeling unwell with vomiting and headache before returning to sleep.

07:30: Family, unable to wake her, called the London Ambulance Service (LAS). The family were all asymptomatic.

08:16: LAS arrived. Examination at the scene found the patient to be tachypnoeic at 40 breaths per minute, tachycardic at 123 beats per minute and with a Glasgow Coma Scale score of 11 (E3, V3, M5). Further findings included a blood sugar of 10.1 millimoles per litre (mmol/L), diaphoresis, pallor, hypothermia (36.1 degrees Celsius), shivering and miosis. Fasciculations were noted but she had no focal neurology. LAS administered 400 µg naloxone intramuscularly with no response and transported via blue light to A&E.

09:20: Assessment in the resuscitation area by A&E Consultant had the same findings and revealed OP poisoning. Additional pertinent signs included fasciculations in the upper chest and increased secretion load. An electrocardiogram revealed sinus tachycardia and venous blood gas showed lactate of 3.8mmol/L with normal pH and bicarbonate levels.

10:00: The toxicology consultant from the National Poisons Information Service (NPIS) suggested the following: measure serum and red cell cholinesterase levels; hold atropine unless high secretion load and also pralidoxime unless repeated atropine use; consider early intubation due to neuromuscular junction (NMJ) involvement; implement decontamination and donning of surgical mask, visor, gloves and apron.

10:20: The patient continued to exhibit widespread fasciculations, confusion, and tachycardia with a heart rate of 120 bpm. Oxygen saturations were 92% on room air, and her blood pressure was 99/65 millimetres of mercury (mmHg). Oxygen was administered via a Mapleson C circuit before induction using fentanyl (1 microgram per kilogram) and 2 mg/kg of ketamine and rocuronium. A grade 1 view on laryngoscopy showed fully relaxed vocal cords.

14:00-18:00: The patient remained in A&E for a prolonged period of time due to bed availability. Frequent suctioning of thick, yellow secretions was required. She remained tachycardic, especially during fasciculations, and developed hypertension (170/100mmHg). For sedation, she required propofol 340mg/hr, and boluses of fentanyl and midazolam before starting a morphine infusion.

**Figure 1:**
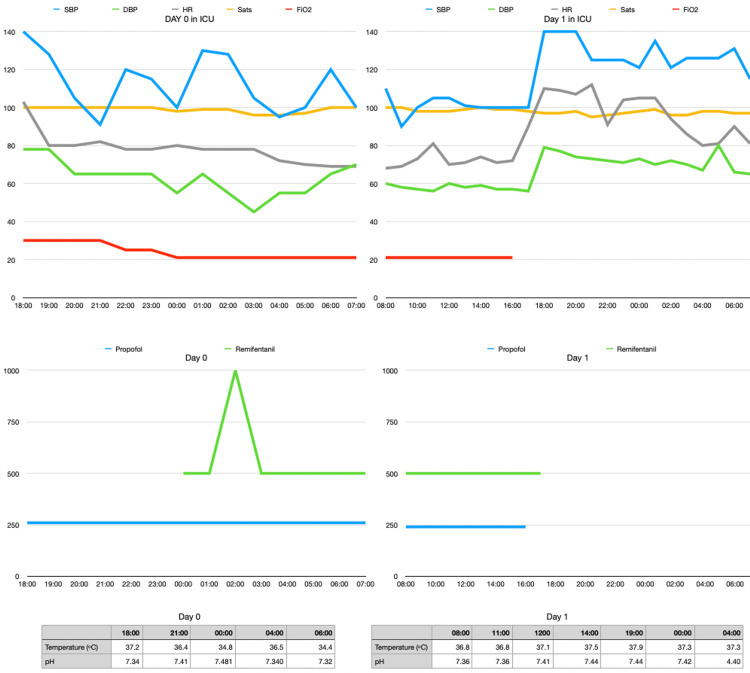
Trends of patient observations during ICU admission

18:30: Admitted to ICU on 30% inspired oxygen (FiO2). Her blood pressure settled to 100mmHg systolic. Her pupils were 2 millimetres and reactive. She experienced recurrent two-minute episodes of fasciculations. Figure 1 shows ICU observations.

Day 1 of admission

02:00: The fasciculations decreased in frequency, haemodynamics stabilised and regular respiratory efforts started.

10:00 Following the ICU ward round, she was extubated and discharged the following day.

Blood results from the admission are seen in Table 1.

**Table 1:** Patient's blood results from admission

	Day 0	Day 1	Day 2	Reference Range
Haemoglobin (grams/litre)	136	126	125	115-165
White Blood Cells (x10^9^/Litre)	11.1	10.4	8.8	3.6-11.0
Platelets (x10^9^/Litre)	194	159	149	140-400
Neutrophils (x10^9^/Litre)	9.1	8.0	6.7	1.8-7.5
International Normalised Ratio (INR)	1.1	1.1	1.2	0.8-1.1
Prothrombin Time (PT) (seconds)	11.8	11.9	12.7	9-13
Sodium (millimoles/litre)	142	142	142	135-146
Potassium (millimoles/litre)	4.4	4.5	3.8	3.5-5.3
Chloride (millimoles/litre)	113	113	109	98-106
Creatinine (micromoles/litre)	87	71	69	45-84
Albumin (grams/litre)	36	34	34	35-50
Total Protein (grams/litre)	60	56	60	60-93
Calcium Adjusted (millimoles/litre)	2.11	2.04	2.21	2.2-2.6
CRP (milligrams/litre)	7	51	87	<5
Creatine Kinase (units/litre)	128	133	381	25-200
Magnesium (millimoles/litre)	0.8	1.2	0.8	0.7-1.0

Follow-up after one month

The patient was fatigued for three days following discharge but has now made a full recovery. She is back at work. Serum anti-cholinesterase levels were very low (<1000 units/litre) in keeping with severe OP poisoning.

## Discussion

Other cases report non-accidental ingestion of OP with 30% of suicide cases globally due to OP intoxication [9] and most patients received atropine and pralidoxime. A UK-based report treated with atropine and intubated achieved a similar recovery to this patient, without pralidoxime [10]. One paper suggested OP poisoning mortality rates ranged from 3% to 25% worldwide [1]. The UK NPIS report 2023 found, from 579 cases of pesticide poisoning, 81.3% were unintentional acute cases and only 11% of cases were graded severe, which this patient would be categorised into [11].

Muscle relaxant

Ketamine 2 mg/kg due to the patient’s haemodynamic instability and fentanyl 1 mcg/kg were administered. The challenging discussion was the type and dose of muscle relaxant. Suxamethonium is a more potent agonist with greater affinity for the cholinergic receptors due to its structure of two acetylcholine (ACh) molecules joined. Its effects will be exacerbated in the presence of a cholinesterase inhibitor [12]. It is contraindicated due to the risk of prolonged respiratory paralysis [13] and extra junctional receptor side effects following muscle injury suggested by a rising creatine kinase, such as hyperkalemia. Rocuronium at 2 mg/kg achieved suitable vocal cord relaxation within 45 seconds of administration. An increased dose was necessary to overcome increased ACh at the NMJ. In the unlikely event that extubation was required, sugammadex could be considered as a reversal agent. It would be interesting to assess the level and type of muscular blockade prior to and post administration of muscle relaxant using a train-of-four nerve stimulator. This could be tested in a future similar situation if the patient is in a condition where pain from stimulation will not be felt, such as post-induction and pre-muscle relaxant, if starved and a modified rapid sequence induction is justified.

Antidotes

The toxicology advice was to administer atropine only if there was high secretion load or severe bradycardia, due to the risk of worsening agitation, and initially, pralidoxime was recommended as per ToxBase guidelines; however, later re-advised to hold due to the risk of hypotension and limited evidence. The mainstay of treatment for this patient was intubation and ventilation followed by supportive level 3 care in the ICU. It is interesting that despite an anti-cholinesterase activity of <1000 units/litre, no antidote was required in this patient. The authors question whether the type of OP may affect the nicotinic and muscarinic receptors differently and therefore the traditional treatment should be tailored to the receptor-type effects, as in this case. Muscarinic effects include SLUDGE symptoms (salivation, lacrimation, urination, defecation, gastric upset and emesis) and bradycardia whilst nicotinic effects include skeletal muscle contraction, weakness, cramps, tachycardia and seizures [14]. The intermediate syndrome has a varying level of severity and often is undiagnosed unless significant respiratory insufficiency. The fatigue symptoms reported in the days following discharge by this patient may have been the effects of mild intermediate syndrome, and further suggestion of nicotinic involvement [15].

## Conclusions

This case study demonstrated the importance of proactive supportive management for patients with OP poisoning and underscored the need for early intubation when NMJ involvement is suspected. Unlike other published case reports, no "traditional" antidote was given in this case of severe OP poisoning with an anticholinesterase level <1000 units/litre. The patient's condition improved within two days after which she was discharged home. There were no staff symptoms from contamination following appropriate personal protective equipment (PPE) and decontamination. Intubation was easy and successful with an increased dose of muscle relaxant. Further investigation of the type and density of neuromuscular blockade in the future may aid in further tailoring of muscle relaxant dosing as opposed to a significantly increased dose. The symptoms and signs exhibited by this patient may suggest the varying effects of OP on the ACh receptors, specifically nicotinic rather than solely muscarinic symptoms.
